# Anesthesiology Handoff Simulation Case: A Handoff From Intensive Care Unit to Operating Room for Anesthesiology Learners

**DOI:** 10.15766/mep_2374-8265.10887

**Published:** 2020-03-13

**Authors:** Sandeep Krishnan, Nakul Kumar, Erik Diaz, Imani Thornton, Farhad Ghoddoussi, Terry A. Ellis

**Affiliations:** 1 Associate Professor, Department of Anesthesiology, Wayne State University School of Medicine; 2 Program Director, Department of Anesthesiology, Wayne State University School of Medicine; 3 Chief of Cardiothoracic Anesthesiology, Department of Anesthesiology, Wayne State University School of Medicine; 4 Resident Physician, Department of Anesthesiology, The Cleveland Clinic; 5 Resident Physician, Department of Anesthesiology, Wayne State University School of Medicine; 6 Assistant Professor, Department of Anesthesiology, Wayne State University School of Medicine; 7 Research Associate, Department of Anesthesiology, Wayne State University School of Medicine

**Keywords:** Handover, Anesthesia, Simulation, Anesthesiology, Patient Handoff

## Abstract

**Introduction:**

Handoffs have been shown to be a potential cause of communication failures, leading to possible inefficiencies and patient harm. We noticed that our CA-1 residents were struggling with patient handoffs and designed this simulation to improve their handoff skills.

**Methods:**

This anesthesiology-specific simulation introduced learners to the perioperative handoff process. We designed it for anesthesiology learners, including junior residents, medical students, and student nurse anesthetists. The simulation centered upon an anesthesiology resident taking care of an ICU patient and handing that patient off to another anesthesiology provider, who took the patient to the OR. We charged learners with reviewing the patient's history and hospital course and giving a complete handoff. We evaluated learners on the completeness and quality of the handoff, as well as on their performance during the session.

**Results:**

Twenty-seven learners participated in this handoff simulation. The participants reported that the simulation improved their understanding of the anesthetic implications of medical conditions and gave them a better understanding of the essential elements of a handoff. Learners also indicated that the debriefing portion of the simulation was effective in filling some of their medical knowledge gaps and improving their handoff skills.

**Discussion:**

This simulation was found to be an effective educational experience for our CA-1 and CA-3 residents, medical students, and student nurse anesthetists. Feedback was positive from all learners. As a result, this simulation will be implemented in the early learning curriculum for all of our CA-1 residents.

## Educational Objectives

At the end of this activity, learners will be able to:

1. Examine patient data and identify information that may impact anesthetic care.
2. Deliver a concise and complete handoff that covers the significant points of a patient's health history and all pertinent anesthetic implications.
3. Communicate the handoff efficiently and effectively to maximize the exchanged information.
4. Describe the elements of patient handoff that are essential to quality perioperative care.

## Introduction

In anesthesia, there are at least three typical scenarios where handoffs between providers are necessary: end-of-shift relief, breaks, and transitions of care. Data have shown that these handoffs are times of significant risk to patients.^1^ Specifically, studies have shown that handoffs from OR to postanesthesia care unit (PACU) and from OR to ICU often are laden with inaccuracies and miscommunication.^2,3^ Three recent studies about handoffs and patient outcomes reported that there was a significant association between handovers and harm to patients.^4–6^ The largest of the studies found that each anesthesiology care transition was associated with increased odds of in-hospital mortality and major complications.^6^

Handoffs between anesthesia providers are unavoidable due to duty hour restrictions for residents and untoward consequences of provider fatigue.^7^ In addition, a few studies have reported potential benefit to patient handoffs, including detecting errors not noted by one provider, distributing cognitive work associated with patient care, and creating opportunities for social interaction.^8,9^

We observed that our residents faced challenges in conducting effective patient handoffs, including omission of critical information, failure to recognize anesthetic implications of missing information, and inability to emphasize the most pertinent details. Several of these observations have been documented in the literature.

Thus, to address the problem of ineffective handoffs in an experiential way that authentically reflected residents' handoff tasks, we designed a simulation case whereby residents would be required to evaluate a complex patient and provide a handoff to a peer. The simulation was developed with the help and advice of two anesthesiology residents (one CA-1 and one CA-3). Selecting residents from differing training years provided a balanced perspective on the challenges faced by novices that was informed by the insight of more experienced residents.

A significant amount of research attests to how handoffs should be taught. *MedEdPORTAL* has many such publications designed to improve the quality of handoffs spanning many different specialties. These publications include simulations, educational curricula featuring handoff techniques, assessment tools, and even video workshops. O'Toole et al.^10^ and Eskildsen et al.^11^ have published handoff-related resources in *MedEdPORTAL* that can be used by physicians to improve clinical handoffs. Elsewhere, Karamchandani et al.^12^ recently published an article about standardizing a handoff for transfer of care from the ICU to the OR. They reported that a standardized patient handoff can improve patient safety and communication between providers. Although there is significant research on the benefits of handoffs in medicine, there are very few publications on simulation and anesthesiology-specific handoffs, particularly ICU-to-OR handoffs. Our simulation addresses this lack of training with regard to ICU-to-OR handoffs.

## Methods

### Development

We developed this simulation for anesthesiology learners of all levels but primarily for CA-1 residents, medical students, and student nurse anesthetists. The case was developed to increase their experience with giving handoffs and to teach specific information critical to a complete handoff. The curriculum included the simulation case template (Appendix A), learner case template (Appendix B), scoring key (Appendix C), teaching points (Appendix D), and learner evaluation (Appendix E). The simulation case template included primary learning objectives, critical actions, initial presentation of the patient, hospital course and current physical examination of the patient, and ideal scenario flow, as well as anticipated handoff mistakes/omissions. We designed the scenario assuming that our learners would have at least a basic understanding of the potential anesthetic implications of common medical problems. The study was determined to have exempt status by the Saint Joseph Mercy Oakland Institutional Review Board.

The simulation was a 1-hour session evaluating an individual anesthesiology learner. It could be supervised by one or more faculty members. The simulation assessed the learner's ability to evaluate and interpret a complex medical history, as well as the resultant anesthetic implications, and to provide a complete and accurate handoff to another anesthesiology provider. The learner received feedback on the handoff from facilitators and then had the opportunity to evaluate the session.

### Equipment and Environment

The simulation did not require a specific location or any additional simulation equipment other than a pencil or pen, a piece of blank paper to take notes as needed, and the learner case template (Appendix B). The learner had 10 minutes to review the material. Then, the learner gave the oral handoff report to the listener, with the facilitators evaluating the handoff using the scoring key (Appendix C). A quiet, private location was used to avoid interruption. The facilitators also used the scoring key to record notes related to handoff delivery that could be discussed during the debriefing following completion of the simulation.

### Personnel

The simulation allowed for a diversity of learners and was customizable for different types of learners and for locations where it might be administered. It was implemented with at least three participants: the facilitator (faculty member), the learner, and a listener playing the part of the anesthesiology resident physician receiving the handoff report. If a second faculty member or evaluator was available, he or she acted as an additional facilitator to give more reliability to the evaluation data. The facilitator judged the quality of the presentation, and any verbal or nonverbal input from the listener/receiver would have interfered with that process. Technically, a facilitator could have acted as the listener, but we felt strongly that the facilitator taking notes and scoring might be a distraction to the learner. Our facilitators were familiar with the handoff process, had reviewed the evaluation form, and were qualified to provide learner feedback.

### Implementation

In this simulation (see Appendix A), we assigned an anesthesiology learner the role of an anesthesiology resident physician rotating through the ICU, faced with handing off a complex patient to another anesthesiology resident who was taking the patient to the OR for urgent surgery. The simulated patient originally presented to the emergency department with a subdural hematoma after a fall at home. The patient had many comorbidities and, over the course of a 14-day ICU stay, suffered multiple medical complications, underwent procedures, and was ultimately scheduled for an urgent laparotomy.

We provided the learner with the learner case template (Appendix B) at the start of the simulation. This template featured an overview of the simulation, the primary learning objectives, the patient's health history and hospital course, and relevant labs and testing, including the results of procedures. We tasked the learner with reviewing the learner case template for 10 minutes and then giving a thorough handoff report in the subsequent 10 minutes. The 10-minute duration of the case review and 10-minute duration of the handoff were determined by the simulation designers to be a reasonable amount of time to perform a thorough chart review and physical examination on this patient and a realistic amount of time to then give a handoff. The learner reviewed the patient's full history and ICU course, organized the portions of the patient's history that had anesthetic implications (the learner could write notes on a piece of paper), and verbally reported these issues to the resident receiving the handoff. This handoff was evaluated by two facilitators. Using the scoring key (Appendix C), the facilitators scored the learner both qualitatively and quantitatively, which allowed the facilitators to provide targeted feedback to the learner and to review the experience following the simulation.

The teaching points (Appendix D) provided the facilitators with information about the topics the learner should have included in the handoff. The teaching points were reviewed by the facilitators both prior to the simulation and then with the learner after the simulation to fill remaining knowledge gaps.

The learner evaluation (Appendix E) included five questions that allowed participants to give feedback about the simulation to the facilitators. The evaluation was given to learners immediately following the debriefing session.

### Assessment

Created as a learning experience in completing comprehensive and content-appropriate handoffs in the perioperative environment, this simulation was designed for anesthesiology students. The quantitative portion of the scoring key evaluated them on presenting the necessary patient information and assimilating and understanding the medical issues that had anesthetic implications. The qualitative portion of the scoring key gave learners feedback on the effectiveness and appropriateness of the handoff using the Accreditation Council for Graduate Medical Education (ACGME) core competencies, including patient care and procedural skills, practice-based learning and improvement, professionalism, and interpersonal and communication skills. This qualitative portion used a 4-point scale (1 = *requires constant guidance*, 4 = *ready to transition into independent practice*). Both the qualitative and quantitative sections were developed by experienced teaching faculty, reviewed by two experts in anesthesiology, and approved by the simulation steering committee. The simulation could also be used as an assessment tool and a measure of handoff competency.

### Debriefing

Immediately after completing the simulation, a debriefing took place that included the following parts:

- Open-ended questions by facilitators: Early in the debriefing, the facilitators gave the learner the opportunity for self-reflection. Facilitators asked open-ended questions, including “How do you feel the simulation went?” and “Do you feel that this simulation will help you to improve your handoffs in the immediate future?” The learner offered verbal feedback at this time. After receiving the learner's initial feedback, the facilitators showed the learner the scoring key and explained how the simulation was scored. The facilitators also counseled the learner on methods to potentially improve the qualitative aspects of the handoff.
- Didactic review: The facilitators next provided a thorough review and analysis of the topics that had been discussed by the learner and those that had been omitted. This discussion included relevant data clarifying the anesthetic implications each topic had for the patient (Appendix D).
- Learner evaluation: The learner received a five-question evaluation at the conclusion of the debriefing (Appendix E). This offered the learner the opportunity to give feedback on the simulation, including suggestions for improvement. Evaluations were anonymous, so respondents were free to give feedback without fear of repercussion.

## Results

A total of 27 learners participated in the handoff simulation: six CA-1 anesthesiology residents, six CA-3 anesthesiology residents, six medical students rotating in general anesthesiology, three medical students rotating in the surgical ICU, and six student nurse anesthetists. The simulation was run with a CA-2 resident functioning as the anesthesiology resident receiving the handoff and with an anesthesiology faculty member and the lead nurse in the PACU as the facilitators/scorers. The case review and handoff were completed in the allotted times of 10 minutes each in all cohorts.

Scoring sheets were reviewed with participants after the scores had been tallied. Table 1 shows the quantitative results from the handoff simulations. Overall, CA-3 residents performed significantly better than the rest of the learners. The six CA-3 residents had an average score of 25.7 (range: 24–27, *SD* = 1.2). The six CA-1 residents had an average score of 21.0 (range: 17–25, *SD* = 2.8). The group of six student nurse anesthetists had an average score of 20.0 (range: 18–24, *SD* = 2.3). The six non-ICU medical students had an average score of 15.7 (range: 14–19, *SD* = 2.7), whereas the three ICU medical students had an average score of 16.3 (range: 14–19, *SD* = 2.5).

**Table 1.** Scores of Cohorts: Quantitative Evaluation

Cohort	Number	Minimum	Maximum	Average	*SD*
CA-1	6	17	25	21.0	2.8
CA-3	6	24	27	25.7	1.2
Medical student	6	14	19	15.0	2.7
Medical student (ICU)	3	14	19	16.3	2.5
Student nurse anesthetist	6	18	24	20.0	2.3
All together	27	14	27	20.1	4.2

The second portion of the scoring key assessed learners' performance in four ACGME core competencies: patient care and procedural skills, practice-based learning and improvement, professionalism, and interpersonal and communication skills. We designed a four-item, 4-point scale (minimum = 1, maximum = 4) with a maximum total score of 16 for evaluating the learner's handover from the ICU to the OR. Two sets of evaluations were carried out simultaneously by two independent observers on 27 handoffs (12 anesthesiology residents, nine medical students, and six student nurse anesthetists). The results are shown in Table 2.

**Table 2.** Scores of Cohorts: Qualitative Evaluations

	First Evaluation					Second Evaluation				
Competency and Cohort	*N*	Minimum	Maximum	Average	*SD*	*N*	Minimum	Maximum	Average	*SD*
Patient care and procedural skills										
CA-1	6	2	3	2.3	0.5	6	2	3	2.2	0.4
CA-3	6	2	3	2.8	0.4	6	3	4	3.2	0.4
Medical student	6	2	2	2.0	0.0	6	2	2	2.0	0.0
Medical student (ICU)	3	2	2	2.0	0.0	3	2	2	2.0	0.0
Student nurse anesthetist	6	2	3	2.2	0.4	6	2	3	2.3	0.5
All together	27	2	3	2.3	0.5	27	2	4	2.4	0.6
Practice-based learning and improvement										
CA-1	6	2	3	2.3	0.5	6	2	3	2.3	0.5
CA-3	6	3	4	3.2	0.4	6	3	3	3.0	0.0
Medical student	6	2	2	2.0	0.0	6	2	2	2.0	0.0
Medical student (ICU)	3	2	2	2.0	0.0	3	2	2	2.0	0.0
Student nurse anesthetist	6	2	3	2.2	0.4	6	2	3	2.2	0.4
All together	27	2	4	2.4	0.6	27	2	3	2.3	0.5
Professionalism										
CA-1	6	3	4	3.2	0.4	6	3	3	3.0	0.0
CA-3	6	2	4	3.2	0.8	6	3	4	3.3	0.5
Medical student	6	2	3	2.8	0.4	6	3	3	3.0	0.0
Medical student (ICU)	3	2	3	2.7	0.6	3	2	3	2.7	0.6
Student nurse anesthetist	6	3	4	3.2	0.4	6	3	4	3.5	0.5
All together	27	2	4	3.0	0.5	27	2	4	3.1	0.5
Interpersonal and communication skills										
CA-1	6	2	3	2.7	0.5	6	2	3	2.5	0.5
CA-3	6	3	4	3.7	0.5	6	3	4	3.5	0.5
Medical student	6	2	2	2.0	0.0	6	2	3	2.2	0.4
Medical student (ICU)	3	2	3	2.3	0.6	3	2	3	2.3	0.6
Student nurse anesthetist	6	3	3	3.0	0.0	6	2	4	3.0	0.6
All together	27	2	4	2.8	0.7	27	2	4	2.7	0.7

The first facilitator's qualitative observations (*N* = 27) had an average score of 10.5 (range: 8–15, *SD* = 1.8, *SE* = 0.4). The second facilitator's qualitative observations (*N* = 27) had an average score of 10.6 (range: 8–14, *SD* = 1.8, *SE* = 0.3). To check the internal consistency of the qualitative scale (comprising four items), we carried out a reliability analysis by calculating Cronbach's alpha for both sets of evaluations.^13^ Overall, the average alpha for both sets of evaluations was .8, which reflects good internal consistency for the qualitative scale designed for the anesthesiology handoff.

As another measure of interrater (test-retest) reliability, we calculated the intraclass correlation coefficient (ICC; two-way, random, absolute agreement) between the score values of the two independent observations for each of the four items of the scale.^14^ There was excellent agreement between the two observers for the whole scale (ICC = .93; *p* < .0005; 95% confidence interval, 0.86–0.97).

During the initial debriefing period, the participants reported that there were no issues with the imposed time limits. Ten participants (four CA-1 residents, one CA-3 resident, three medical students, and two student nurse anesthetists) commented on their need to improve their organization during the initial debriefing period, and many participants recognized points that they had missed during the handoff. Twenty-five learners felt that the case was realistic, and all 27 learners agreed that their handoff skills improved as a result of the simulation and debriefing. CA-3 residents specifically stated that they wished they had participated in this simulation during their CA-1 year.

Verbal and written feedback from all groups was consistently good. In terms of written feedback, each learner was given a five-question evaluation that utilized a 5-point Likert scale (1 = *strongly disagree*, 5 = *strongly agree*). Questions 1, 2, 3, and 5 averaged scores above 4 from all groups. CA-1 residents and medical students reported lower scores on question 4 than CA-3 residents and student nurse anesthetists. A few of the CA-1 residents and many of the medical students reported feeling that some of the information presented in the clinical scenario was above their level of knowledge. Many learners reported that the didactic review was beneficial in filling their knowledge gaps, and some learners asked to have a written copy of the didactic review to peruse at a later time.

Responses to our open-text questions demonstrated several themes. Residents commented on the authenticity of the simulation:

- “Great simulation! Felt like I was in the ICU.”
- “Great complex simulation!”

They also found the simulation to be useful and enjoyable:

- “This was a great exercise for residents of all levels. It is a complex case and has utility for even CA-3 residents.”
- “I enjoyed this simulation immensely!”
- “Very useful simulation for CA-1 residents!”

All participants had positive feedback for the simulation and stated that they improved their understanding of the perioperative anesthesiology handoff process during the session.

## Discussion

To improve the quality of handoffs among our anesthesiology residents, we created a simulation case that allowed learners to practice handoffs without harming actual patients and to receive feedback through debriefing on the clinical scenario.

Our simulation of anesthesiology-specific handoffs from the ICU to the OR was well received by learners overall. The complex case had multiple pitfalls, many of which could easily be overlooked during a handoff process. The complexity of the scenario was noted and appreciated by the learners. They felt that it gave them an opportunity to review a large amount of information, condense and absorb it, and provide a summative review of a very sick patient. A majority of the residents reported feeling that the clinical scenario was appropriate to their level of training and relevant to their daily practice. Three medical students felt that the clinical scenario was well above the level of their training. All of our learners did report, however, that they appreciated and enjoyed the simulation. We did not use the simulation as a formal assessment tool for our participants' handoff skills, but it has great potential to be used as such a tool.

The scoring key was based in part on core competencies created by the ACGME. These core competencies define the skills that the ACGME believes every practicing physician should possess. Our department's grading and evaluation tools use the core competencies to evaluate residents. The qualitative scoring key (see Appendix C) showed good internal consistency and good interrater reliability.

During implementation of the simulation, we faced a few challenges. The first was finding a time when both the faculty facilitator and the PACU lead nurse facilitator were available. Running over the course of 7 separate days, the simulation necessitated a significant time commitment from both the faculty facilitator and the PACU lead nurse facilitator. Additionally, we struggled to arrange for learners and listeners to be free from clinical duties so that they could participate in the simulation. With careful planning, we were able to find times when all parties were available. One potential solution for future uses would be to shorten the time for each simulation. Although scheduled for 1 hour, the simulation could be shortened considerably by decreasing the length of the debriefing session and instead providing all learners with a copy of the teaching points to peruse at their leisure. The largest challenge that we faced, however, was the learners having difficulty staying in character. They would occasionally look to the facilitators for guidance or to ask them questions. However, we instructed the facilitators to stay silent, and they strictly upheld that rule. In the future, more explicit instructions will be given to learners prior to participating in the simulation.

A potential limitation to our simulation was our inability to fully evaluate its impact. Our evaluation did not give us the ability to demonstrate any behavioral change among the learners in the clinical setting. In the future, we will create an additional case scenario for learners to complete a few months later to determine whether they are truly learning and actually changing their behavior. Another limitation to our simulation was that we did not consider that student nurse anesthetists do not provide care in the ICU and therefore would not be handing off care from the ICU to the OR. However, we did not receive any negative feedback from student nurse anesthetists about the simulation not being relevant to their clinical practice (question 2 on the learner evaluation). In fact, several student nurse anesthetists verbally reported that although they did not participate in ICU care anymore, “the anesthetic implications of a sick patient were certainly relevant” to them and they “appreciated the additional education.” Another potential limitation to the simulation was that our listeners did not give feedback to or ask questions of the learners. In most handoffs, there is a dialogue between the practitioner giving the handoff and the practitioner receiving the handoff. This dialogue often results in a more complete handoff. In the future, we may change the simulation to allow the listener to ask the learner pertinent questions to elicit salient information.

Because of the positive feedback that we received, we implemented this simulation as a standard handoff session for all of our CA-1 residents in their first few months of training. In repeating the simulation this year, however, we realized that oftentimes the ICU-to-OR handoff is given by someone who is not an anesthesiology practitioner. This simulation has the potential to educate other practitioners who participate in ICU-to-OR handoffs, including internal medicine residents, surgery residents, nurse practitioners, and even physician assistants. We are working with our surgery residency faculty to incorporate this anesthesiology-specific handoff into their PGY-1 training. Overall, this simulation met all of our educational objectives and is an enjoyable session that can be used with trainees. We are in the process of creating additional clinical scenarios with the goal of having multiple handoff simulation sessions during the CA-1 year and measuring incremental performance change over that time period among our learners.

## Appendices

A. Simulation Case.docx
B. Learner Case.docx
C. Scoring Key.docx
D. Teaching Points.docx
E. Learner Evaluation.docx All appendices are peer reviewed as integral parts of the Original Publication.
