# Supplementary material for: Anesthesiology Handoff Simulation Case: A Handoff From Intensive Care Unit to Operating Room for Anesthesiology Learners
Source: MedEdPORTAL. 2020 Mar 13;16:10887. doi: 10.15766/mep_2374-8265.10887 (PMC7083603; doi:10.15766/mep_2374-8265.10887)
Supplement: Supplementary file 1 — A. Simulation Case.docx B. Learner Case.docx C. Scoring Key.docx D. Teaching Points.docx E. Learner Evaluation.docx [file mep-16-10887-s001.zip › E. Learner Evaluation.docx]

Appendix E

**Learner Evaluation**

I am a: (Circle One)

Medical Student Anesthesiology Resident Student Nurse Anesthetist

1= strongly disagree 3= neutral 5= strongly agree

1. The simulation was well designed to meet the educational objectives

1 2 3 4 5

1. The scenario was relevant to my clinical practice

1 2 3 4 5

1. The simulation and debriefing session improved my understanding of the anesthetic implications of medical conditions

1 2 3 4 5

1. The simulation was at an appropriate level for my medical knowledge

1 2 3 4 5

1. The simulation was beneficial and enjoyable

1 2 3 4 5

Comments: ­­­­­­­­­­­­_______________________________________________________________________________________________________________________________________________________________________________________________________________________________________________________________________________________
