# Supplementary material for: Anesthesiology Handoff Simulation Case: A Handoff From Intensive Care Unit to Operating Room for Anesthesiology Learners
Source: MedEdPORTAL. 2020 Mar 13;16:10887. doi: 10.15766/mep_2374-8265.10887 (PMC7083603; doi:10.15766/mep_2374-8265.10887)
Supplement: Supplementary file 1 — A. Simulation Case.docx B. Learner Case.docx C. Scoring Key.docx D. Teaching Points.docx E. Learner Evaluation.docx [file mep-16-10887-s001.zip › A. Simulation Case.docx]

| **Appendix A: MedEdPORTAL Simulation Case Template**  **SIMULATION CASE TITLE:** Anesthesiology Handoff Simulation: An Intensive Care Unit to Operating Room Handoff for Anesthesiology Learners  **AUTHORS**: Sandeep Krishnan, MD, Nakul Kumar, MD, Erik Diaz, MD, Imani Thornton, MD, Farhad Ghoddoussi, PhD, Terry Ellis, II MD | |
| --- | --- |
| **PATIENT NAME:** LK  **PATIENT AGE:** 77-year-old female  **CHIEF COMPLAINT:** Fall with subdural hematoma | |
|  | |
| **Brief narrative description of case** | You are on-call in the Intensive Care Unit (ICU). You have been taking care of a patient that has been in the ICU for 14 days. Today, the surgical team decides to take your patient to the Operating Room for an urgent laparotomy. You are tasked to give a thorough handoff to the anesthesiology resident who does not know this patient but will be taking care of the patient in the operating room. The goals for the case are to give a complete and concise handoff to the anesthesiology resident which will allow them to take care of the patient safely. |
| **Primary learning objectives** | 1. Examine patient data and identify information that may impact anesthetic care. 2. Deliver a concise handoff completely covering the significant points of a patient’s health history. 3. Use written and verbal communication skills to completely transfer pertinent information to care teams. 4. Describe the elements of patient handoff that are essential to quality perioperative care. |
| **Critical actions** | 1. Describe procedure 2. Give a complete past medical history of the patient 3. Describe any current neurologic deficits that the patient may have 4. Discuss ventilator settings 5. Discuss airway status-tracheostomy/endotracheal tube (ETT) (cuffed vs.uncuffed), concern with airway edema, fluid overload 6. Discuss NPO status 7. Discuss cardiac stenting-BMS/DES, anticoagulation (specific drug and last dose), pertinent coagulation labs 8. Describe pacemaker/ICD settings 9. Discuss patient’s current cardiac rhythm 10. Detail patient’s current medications-specifically beta blocker, ACEi, diuretics 11. Discuss recent cardiac studies-echocardiogram including left ventricular ejection fraction (LVEF) and valve pathology 12. Discuss transfusions-blood products transfused during hospitalization, type and screen completed for OR, mention of pulmonary congestion early in admission after administration of fluids/transfusion 13. Discuss recent laboratory values including electrolytes and recent ABG 14. Discuss current blood glucose and treatment plan 15. Discuss dialysis-time of last dialysis, volume of fluid removed 16. Discuss antibiotics-scheduled antibiotics and administration time 17. Discuss patient’s vascular access-peripheral and central IV access, invasive monitoring 18. Complete the handoff in 10-minute time limit |
| **Learner preparation** | Learners should ideally have at least some exposure to anesthesiology and the perioperative process and should have a basic understanding of the anesthetic implications of medical issues arising in a hospital setting. Some ICU experience is also preferred, but not required. |

| Initial Presentation | | | |
| --- | --- | --- | --- |
|  |  | | |
| **Overall appearance** | Learners are presented with the case template when they enter the room. | | |
| **Actors and roles in the room at case start** | At case start, the listener (role-playing receiver of the report) and at least one facilitator is present in the room. The listener can be played by almost anyone. The listener does not interact or give any feedback to the learner, they only listen. In our simulation, the listener was played by an anesthesiology resident. This simulation is ideally run with two facilitators. The facilitator is played by an anesthesiology faculty member who is familiar with the simulation. A second facilitator aids with evaluating the learner, who was played by the lead nurse in the PACU for our simulation. However, this simulation can be run with only one facilitator as well. | | |
| **HPI** | A 77-year-old female, LK, was admitted to the emergency department (ED) two weeks ago after a fall in which she hit her head. LK has a past medical history of hypertension (HTN), diabetes mellitus type 2 (DM2), coronary artery disease with remote history of stent placement, atrial fibrillation (A-fib), congestive heart failure (CHF), hyperlipidemia and chronic obstructive pulmonary disease (COPD). The patient was obtunded at the time of examination and intubated in emergency department for airway protection. CT scan obtained showed a moderate-sized subdural hematoma without a midline shift. Neurosurgery was consulted, and the patient was admitted to the Surgical Intensive Care Unit (SICU) for close monitoring and reversal of anticoagulation. Below is her Past Medical/Surgical History, Medications, Allergies, Family History, and Imaging ON ADMISSION. | | |
| **Past medical/surgical history** | **Medications** | **Allergies** | **Family history** |
| PMHx:1. Coronary artery disease status post drug-eluting stents (DES) X 2 placed in left anterior descending coronary artery (LAD) and circumflex coronary artery (LCx) 7 years ago  - 2. CHF with last left ventricular ejection fraction (LVEF) 35% on transthoracic echo 4 weeks ago - 3. HTN - 4. Hyperlipidemia - 5. DM2 on long acting insulin - 6. COPD with emphysema, 2 pack per day smoker, quit in 2010 - 7. Atrial fibrillation - 8. Gastroesophageal reflux disease (GERD) - 9. Chronic kidney disease (CKD) stage 2  PSHx:  - 1. Appendectomy - 2. Cholecystectomy - 3. Exploratory laparotomy for bowel obstruction in 1997 | - Aspirin 81 mg PO daily - Warfarin 7.5 mg PO daily - Atorvastatin 40mg PO daily - Lisinopril 20 mg PO daily - Metoprolol 25 mg PO BID - Furosemide 20 mg PO in morning - Potassium Chloride 20 mEq PO daily - Omeprazole 20 mg PO at bedtime - Albuterol/ipratropium 2 puffs Q4 hrs PRN - Budesonide 0.5 mg inh once daily | - No known drug allergies | Unknown |

| **Imaging on admission** | Chest X-ray: Hyperinflated lung fields, mild congestion, small bilateral pleural effusions.  ETT 2.5cm above the carina, nasogastric tube (NGT) tube tip seen in proximal stomach.  CT head: small to moderate sized left sided subdural hematoma without midline shift or significant mass effect. |
| --- | --- |

**Patient hospital course**

After admission, the patient had a complicated 14-day hospital course. The pertinent details of that hospital course are described below. You are tasked with presenting the important information to the resident taking the patient from the ICU to the OR. Current physical exam is listed after the clinical course.

## Day 1:

Head CT repeated and no further bleeding seen with stable subdural hematoma. Desmopressin given for suspected central diabetes insipidus. Additional 3 liters of crystalloid given to compensate for large urine output. Reduction in urine output seen after desmopressin with an increase in urine electrolytes. Patient agitated with decreased sedation but moving all extremities. Neurosurgery recommends no surgical intervention at this point. All home meds except anticoagulation and diuretics continued.

## Day 2:

Increased frothy secretions seen from endotracheal tube. Patient’s saturations drop over a few hours from 98% to 84%, and SICU staff unable to increase saturations substantially with 100% FiO2. Chest x-ray reveals significant pulmonary edema and moderate bilateral pleural effusions. ABG shows low PaO2. Low tidal volume ventilation and higher PEEP settings initiated. Furosemide therapy started for fluid overload likely from preexisting CHF. Patient remains hemodynamically stable. Improvement in SpO2 to 92% seen. Repeat head CT reveals improvement in hematoma, and neurosurgery signs off, stating it is ok to restart anticoagulation after 5 more days if no signs of bleeding. Tube feeding via NGT started.

## Day 3:

Patient with short 6-beat run of ventricular tachycardia last night. Electrolytes revealed a low potassium of 2.7 mEq/L and magnesium of 1.1 mg/dL, both replaced promptly and defibrillator pads placed on patient. K+ improved to 3.4 mEq/L, no additional episodes of ventricular tachycardia seen. Sedation decreased again today, patient more reactive and moving all extremities, but still not following commands.

## Day 4:

Patient’s SpO2 is not improving beyond 92-94% on 100% FiO2, and increased thick colored sputum seen, which was sent for cultures. Repeat chest x-ray shows consolidation in the right lower lobe of lung. Antibiotic therapy initiated for pneumonia with Vancomycin 1 gram daily and Piperacillin/Tazobactam 3.375 grams every 6 hours. Home diuretic therapy maintained with lower urine output compared to day before.

## Day 6:

Patient is now oliguric despite furosemide therapy overnight. BUN and creatinine show signs of acute renal failure. Potassium level now 4.8 mEq/L up from 3.8 mEq/L yesterday. Increased maintenance fluid therapy. Antibiotics changed to ceftriaxone 1 gram daily and metronidazole 500 mg twice daily. Nephrology consulted, and they relate that patient may require dialysis if acute renal failure (ARF) does not improve.

## Day 8:

Improvement in ventilation and oxygenation seen with a reduction in FiO2 and PEEP. ARF worsening, and a Quinton dialysis catheter inserted at bedside. Potassium increased to 5.4 mEq/L, calcium gluconate and insulin/dextrose therapy given. Patient dialyzed at bedside with removal of 2 liters of fluid.

## Day 10:

Patient received a percutaneous endoscopic gastrostomy (PEG) tube and tracheostomy with 8.0 cuffed Shiley which was placed at bedside, tolerated well. Sedation decreased, patient is now able to follow commands, but does have some right-sided weakness in upper and lower extremities. Developed atrial fibrillation with rapid ventricular response post operatively, and treatment with IV metoprolol was unsuccessful. A diltiazem drip started at 5mg/hr with rate control, however this was discontinued after an hour due to hypotension. Warfarin has been restarted, without bridging therapy.

## Day 11:

Sudden bradycardia seen with heart rate in the 30’s. EKG shows 3^rd^ degree AV block and non-specific ST segment changes. Atropine administered with improvement in HR. Stat bedside transthoracic echo performed which showed LVEF decreased to 25% with hypokinesis of inferior wall, severe tricuspid regurgitation, and moderate right ventricular dilation. Warfarin discontinued and IV heparin therapy started, emergent cardiology consult obtained and cardiac catheterization performed which showed 95% posterior descending artery stenosis. Balloon angioplasty performed and drug-eluting stent placed. In addition, temporary venous pacemaker inserted into right internal jugular vein and patient paced using a VVI mode at 80 beats per minute. Peri-procedural aspirin and clopidogrel were also given.

## Day 13:

Significant hemoglobin drop seen from 9.2 g/dL two days ago to 6.8 g/dL today. 2 units pRBC administered for a target > 8 g/dL. Gastroenterology consulted and clopidogrel not given today, per endoscopist’s orders, due to GI bleed. Aspirin given as scheduled. PEG tube feeding held. Esophagogastroduodenoscopy (EGD) showed active bleeding gastric ulcer along lesser curvature of stomach. Hemostasis achieved by endoscopist using epinephrine and clipping. A pantoprazole drip was started and hemoglobin levels checked Q6 hours. Dialysis performed today due to rising K+, with 2.2 liters removed. Potassium level decreased to 3.4 mEq/L post dialysis.

## Day 14 (Today):

Continual decrease in hemoglobin overnight, Hgb 7.2 g/dL today from 6.6 g/dL despite 2 unit pRBC transfusion. Aspirin and clopidogrel held. Another EGD urgently performed which showed further bleeding from previous ulcer site. Multiple methods for hemostasis attempted and upon use of thermal coagulation, moderately-sized perforation created along lesser curvature of stomach. General surgery was consulted and urgent laparotomy scheduled. Additional 2 units pRBC given with an additional 2 units on hold for OR. 2 units FFP given as well. Patient is currently maintained on vent with FiO_2_ 40%, 5 PEEP, 8ml/kg TV and rate of 12.

| **Vitals** | Temperature 37^o^ C, HR 110, BP 108/64; Ventilator settings: Assist Control, RR 12, TV 450ml, FiO_2_ 40%, 5 cm H_2_O PEEP, SpO_2_ 97% |
| --- | --- |
| **General** | General: Sedated, Tubes/Lines/Drains: 12.0 French x 16cm curved chest tube, right radial arterial line, double lumen Quinton catheter in right internal jugular vein; transvenous pacemaker placed through 8.5 French Cordis in left subclavian vein; 20-gauge peripheral IV in right forearm |
| **HEENT** | 8.0 cuffed Shiley in place |
| **Neck** |  |
| **Lungs** | Non-labored breathing, bilateral wheezing, no rhonchi or rales |
| **Cardiovascular** | Irregular rate and rhythm, temporary venous pacemaker VVI set to 80 beats per minute. Radial and femoral pulses intact |
| **Abdomen** | Abdomen distended and diffusely tender with severe guarding. Hypoactive bowel sounds. PEG tube in place to dependent drainage. |
| **Neurological** | Sedated, Richmond Agitation-Sedation Scale (RASS) -1. Decreased strength in right upper and lower extremities. |
| **Skin** |  |
| **GU** |  |
| **Psychiatric** |  |

| Instructor Notes - Changes and CASE Branch Points  *This section should be a list with detailed description of each step than may happen during the case. If medications are given, what is the response? Do changes occur at certain time points? Should the nurse or other participant prompt the learners at given points? Should new actors or participants enter, and when? Are there specific things the patient will say or do at given times? There are a few examples given, but it is expected that most cases will have many more changes and potential branch points.* | | |
| --- | --- | --- |
| **Intervention/time point** | **Change in case** | **Additional information** |
| Learner enters simulation room and is provided the Learner Case Template (Appendix B) which includes an overview of the simulation, the primary learning objectives, and the case itself. Learner is provided a quiet space with paper and pencil, told they have 10 minutes to review the case and organize their thinking, taking notes as needed. At the 10-minute mark, learner gives a complete handoff to the listener (receiver of handoff), who does not react, comment or ask clarifying questions. The simulation ends when learner states handoff is complete or when the 10-minute time limit has been reached, whichever comes first. |  | No additional information is provided from any source (as noted, listener does not react, comment or ask clarifying questions) |

**Ideal scenario flow**

The scenario begins with the learner entering the room and receiving the case stem, which includes the history and all pertinent information about the patient in this simulation. The learner will then have 10 minutes to peruse the patient’s history and make notes as needed. After 10 minutes, the learner will then give a complete handoff to the listener, giving the listener all of the information that the learner feels is valuable. The listener does not react or give feedback during this process. The facilitators are evaluating the learner’s handoff using qualitative and quantitative methods.

At the conclusion of the 10-minute handoff, the facilitators will ask the learner open-ended questions about the simulation to allow for self-reflection. After receiving that initial feedback, the facilitators will describe the evaluation process, including showing the learner the qualitative and quantitative scoring key and their scores for the simulation. They will counsel the learner on potential improvements to the qualitative aspects of the handoffs, as well as discuss any specific medical issues and anesthetic implications that were missed or presented incorrectly. The facilitators should then present a focused didactic review of the topics (included with simulation) that were missed or presented incorrectly.

Finally, the learners should be given an evaluation at the conclusion of the debriefing. This gives the learners the opportunity to give feedback to the facilitators and creators of the simulation, including suggestions for improvement.

**Anticipated handoff mistakes/omissions**

1. **Failure to mention the indication for the surgical procedure**-The team taking the patient to the OR needs to understand the indication for surgery so they can start to think about their own anesthetic plan.
2. **Failure to discuss neurologic deficits**-It is critical to have an understanding of baseline neurologic deficits so that the patient can be properly managed intraoperatively and properly assessed post-operatively.
3. **Failure to mention ventilator settings in ICU**-Ventilator settings in the ICU should be reported during handoff. The ICU team often has been managing the patient for many days and has an intimate understanding of the ventilator settings that have been used to support the patient.
4. **Failure to describe the patient’s airway status**-The OR team should have an understanding of how or if the patient is already intubated or has a tracheostomy, and if there are any concerns in management.
5. **Failure to describe NPO status**-Even if the patient is currently intubated, the patient may be getting tube feeds, which may need to be stopped prior to non-emergent surgeries. This is dependent on institutional guidelines as well as location of the feeding tube (stomach vs. jejunum). If feeds are not stopped due to the emergent nature of the surgery, it is important that the OR team is aware of this.
6. **Failure to mention previous cardiac interventions and anticoagulation**-Previous cardiac interventions and anticoagulation should be discussed during a handoff. Even if the stents are remote, it is important to know about a patient’s history of stents because of risk of in-stent thrombosis and potential cardiac issues. There are also management guidelines for elective non-cardiac surgery after stent placement, including managing anti-platelet drugs/anticoagulation. Recent doses of anticoagulants/anti-platelet agents should also be known to prepare for potential intra-operative bleeding.
7. **Failure to mention ICD/pacemaker settings**-Brand of ICD/pacemaker, reason for ICD/pacemaker placement, and settings should be part of a routine handoff. Pacemaker settings can be changed prior to surgery (need to know brand), or a magnet can be placed over the device to temporarily change the way that the pacemaker/ICD works.
8. **Failure to discuss important anti-hypertensive medications**-There is significant data on use of beta blockers, ACE Inhibitors, and ARBs on hemodynamics during surgery and post-operative outcomes. Patients taking beta blockers, chronically, should take their beta blockers on the day of surgery unless there is a specific contraindication. There is conflicting evidence on the use of ACE Inhibitors and ARBs on the day of surgery. These issues need to be discussed with the OR team.
9. **Failure to discuss cardiac function**-If available, it is important to discuss recent echocardiographic findings in patients who are critically ill or going to the operating room.
10. **Failure to discuss recent laboratory values**-Pertinent or abnormal laboratory values should be discussed prior to going to the OR.
11. **Failure to discuss recent dialysis**-Intraoperative fluid management will be influenced by timing of last dialysis and fluid removed.
12. **Failure to mention antibiotics**-Current antibiotic management of patients should be mentioned, and also antibiotics must be administered in a timely fashion if needed for surgical procedures. Data has shown this to be important for patient safety and outcomes.
