# Supplementary material for: Anesthesiology Handoff Simulation Case: A Handoff From Intensive Care Unit to Operating Room for Anesthesiology Learners
Source: MedEdPORTAL. 2020 Mar 13;16:10887. doi: 10.15766/mep_2374-8265.10887 (PMC7083603; doi:10.15766/mep_2374-8265.10887)
Supplement: Supplementary file 1 — A. Simulation Case.docx B. Learner Case.docx C. Scoring Key.docx D. Teaching Points.docx E. Learner Evaluation.docx [file mep-16-10887-s001.zip › B. Learner Case.docx]

Appendix B

Learner Case Template

**Overview**

You are on-call in the Intensive Care Unit (ICU). You have been taking care of a patient that has been in the ICU for 14 days. Today, the surgical team decides to take your patient to the Operating Room for an urgent laparotomy. You are tasked to give a thorough handoff to the anesthesiology resident who does not know this patient but will be taking care of the patient in the operating room. The goals for the case are to give a complete and concise handoff to the anesthesiology resident which will allow them to take care of the patient safely. You will have 10 minutes to review the case and take notes, followed by 10 minutes to give a complete handoff to the listener.

**Primary Learning Objectives**

1. Examine patient data and identify information that may impact anesthetic care.
2. Deliver a concise handoff completely covering the significant points of a patient’s health history.
3. Use written and verbal communication skills to completely transfer pertinent information to care teams.
4. Describe the elements of patient handoff that are essential to quality perioperative care.

**Case**

A 77-year-old female, LK, was admitted to the emergency department (ED) two weeks ago after a fall in which she hit her head. LK has a past medical history of hypertension (HTN), diabetes mellitus type 2 (DM2), coronary artery disease with remote history of stent placement, atrial fibrillation (A-fib), congestive heart failure (CHF), hyperlipidemia and chronic obstructive pulmonary disease (COPD). The patient was obtunded at the time of examination and intubated in emergency department for airway protection. CT scan obtained showed a moderate-sized subdural hematoma without a midline shift. Neurosurgery was consulted and the patient was admitted to the Surgical Intensive Care Unit (SICU) for close monitoring and reversal of anticoagulation. Below is her past medical/surgical history, medications, allergies, family history, and imaging ON ADMISSION. On the following page you will examine LK’s hospital course.

| **Past medical/surgical history** | **Medications** | **Allergies** | **Family history** |
| --- | --- | --- | --- |
| PMHx:1. Coronary artery disease status post drug-eluting stents (DES) X 2 placed in left anterior descending coronary artery (LAD) and circumflex coronary artery (LCx) 7 years ago  - 2. CHF with last left ventricular ejection fraction (LVEF) 35% on transthoracic echo 4 weeks ago - 3. HTN - 4. Hyperlipidemia - 5. DM2 on long acting insulin - 6. COPD with emphysema, 2 pack per day smoker, quit in 2010 - 7. Atrial fibrillation - 8. Gastroesophageal reflux disease (GERD) - 9. Chronic kidney disease (CKD) stage 2  PSHx:  - 1. Appendectomy - 2. Cholecystectomy - 3. Exploratory laparotomy for bowel obstruction in 1997 | - Aspirin 81 mg PO daily - Warfarin 7.5 mg PO daily - Atorvastatin 40mg PO daily - Lisinopril 20 mg PO daily - Metoprolol 25 mg PO BID - Furosemide 20 mg PO in morning - Potassium Chloride 20 mEq PO daily - Omeprazole 20 mg PO at bedtime - Albuterol/ipratropium 2 puffs Q4 hrs PRN - Budesonide 0.5 mg inh once daily | - No known drug allergies | Unknown |

| **Imaging on admission** | Chest X-ray: Hyperinflated lung fields, mild congestion, small bilateral pleural effusions.  ETT 2.5cm above the carina, nasogastric tube (NGT) tube tip seen in proximal stomach.  CT head: small to moderate sized left sided subdural hematoma without midline shift or significant mass effect |
| --- | --- |

**Patient hospital course**

After admission, the patient had a complicated 14-day hospital course. The pertinent details of that hospital course are described below. You are tasked with presenting the important information to the resident taking the patient from the ICU to the OR. Current physical exam is listed after the clinical course.

## Day 1:

Head CT repeated and no further bleeding seen with stable subdural hematoma. Desmopressin given for suspected central diabetes insipidus. Additional 3 liters of crystalloid given to compensate for large urine output. Reduction in urine output seen after desmopressin with an increase in urine electrolytes. Patient agitated with decreased sedation but moving all extremities. Neurosurgery recommends no surgical intervention at this point. All home meds except anticoagulation and diuretics continued.

## Day 2:

Increased frothy secretions seen from endotracheal tube. Patient’s saturations drop over a few hours from 98% to 84%, and SICU staff unable to increase saturations substantially with 100% FiO2. Chest x-ray reveals significant pulmonary edema and moderate bilateral pleural effusions. ABG shows low PaO2. Low tidal volume ventilation and higher PEEP settings initiated. Furosemide therapy started for fluid overload likely from preexisting CHF. Patient remains hemodynamically stable. Improvement in SpO2 to 92% seen. Repeat head CT reveals improvement in hematoma, and neurosurgery signs off, stating it is ok to restart anticoagulation after 5 more days if no signs of bleeding. Tube feeding via NGT started.

## Day 3:

Patient with short 6-beat run of ventricular tachycardia last night. Electrolytes revealed a low potassium of 2.7 mEq/L and magnesium of 1.1 mg/dL, both replaced promptly and defibrillator pads placed on patient. K+ improved to 3.4 mEq/L, no additional episodes of ventricular tachycardia seen. Sedation decreased again today, patient more reactive and moving all extremities, but still not following commands.

## Day 4:

Patient’s SpO2 is not improving beyond 92-94% on 100% FiO2, and increased thick colored sputum seen, which was sent for cultures. Repeat chest x-ray shows consolidation in the right lower lobe of lung. Antibiotic therapy initiated for pneumonia with Vancomycin 1 gram daily and Piperacillin/Tazobactam 3.375 grams every 6 hours. Home diuretic therapy maintained with lower urine output compared to day before.

## Day 6:

Patient is now oliguric despite furosemide therapy overnight. BUN and creatinine show signs of acute renal failure. Potassium level now 4.8 mEq/L up from 3.8 mEq/L yesterday. Increased maintenance fluid therapy. Antibiotics changed to ceftriaxone 1 gram daily and metronidazole 500 mg twice daily. Nephrology consulted, and they relate that patient may require dialysis if acute renal failure (ARF) does not improve.

## Day 8:

Improvement in ventilation and oxygenation seen with a reduction in FiO2 and PEEP. ARF worsening, and a Quinton dialysis catheter inserted at bedside. Potassium increased to 5.4 mEq/L, calcium gluconate and insulin/dextrose therapy given. Patient dialyzed at bedside with removal of 2 liters of fluid.

## Day 10:

Patient received a percutaneous endoscopic gastrostomy (PEG) tube and tracheostomy with 8.0 cuffed Shiley which was placed at bedside, tolerated well. Sedation decreased, patient is now able to follow commands, but does have some right-sided weakness in upper and lower extremities. Developed atrial fibrillation with rapid ventricular response post operatively, and treatment with IV metoprolol was unsuccessful. A diltiazem drip started at 5mg/hr with rate control, however this was discontinued after an hour due to hypotension. Warfarin has been restarted, without bridging therapy.

## Day 11:

Sudden bradycardia seen with heart rate in the 30’s. EKG shows 3^rd^ degree AV block and non-specific ST segment changes. Atropine administered with improvement in HR. Stat bedside transthoracic echo performed which showed LVEF decreased to 25% with hypokinesis of inferior wall, severe tricuspid regurgitation, and moderate right ventricular dilation. Warfarin discontinued and IV heparin therapy started, emergent cardiology consult obtained and cardiac catheterization performed which showed 95% posterior descending artery stenosis. Balloon angioplasty performed and drug-eluting stent placed. In addition, temporary venous pacemaker inserted into right internal jugular vein and patient paced using a VVI mode at 80 beats per minute. Peri-procedural aspirin and clopidogrel were also given.

## Day 13:

Significant hemoglobin drop seen from 9.2 g/dL two days ago to 6.8 g/dL today. 2 units pRBC administered for a target > 8 g/dL. Gastroenterology consulted and clopidogrel not given today, per endoscopist’s orders, due to GI bleed. Aspirin given as scheduled. PEG tube feeding held. Esophagogastroduodenoscopy (EGD) showed active bleeding gastric ulcer along lesser curvature of stomach. Hemostasis achieved by endoscopist using epinephrine and clipping. A pantoprazole drip was started and hemoglobin levels checked Q6 hours. Dialysis performed today due to rising K+, with 2.2 liters removed. Potassium level decreased to 3.4 mEq/L post dialysis.

## Day 14 (Today):

Continual decrease in hemoglobin overnight, Hgb 7.2 g/dL today from 6.6 g/dL despite 2 unit pRBC transfusion. Aspirin and clopidogrel held. Another EGD urgently performed which showed further bleeding from previous ulcer site. Multiple methods for hemostasis attempted and upon use of thermal coagulation, moderately-sized perforation created along lesser curvature of stomach. General surgery was consulted and urgent laparotomy scheduled. Additional 2 units pRBC given with an additional 2 units on hold for OR. 2 units FFP given as well. Patient is currently maintained on vent with FiO_2_ 40%, 5 PEEP, 8ml/kg TV and rate of 12.

| **Vitals** | Temperature 37^o^ C, HR 110, BP 108/64; Ventilator settings: Assist Control, RR 12, TV 450ml, FiO_2_ 40%, 5 cm H_2_O PEEP, SpO_2_ 97% |
| --- | --- |
| **General** | General: Sedated, Tubes/Lines/Drains: 12.0 French x 16cm curved chest tube, right radial arterial line, double lumen Quinton catheter in right internal jugular vein; transvenous pacemaker placed through 8.5 French Cordis in left subclavian vein; 20-gauge peripheral IV in right forearm |
| **HEENT** | 8.0 cuffed Shiley in place |
| **Neck** |  |
| **Lungs** | Non-labored breathing, bilateral wheezing, no rhonchi or rales |
| **Cardiovascular** | Irregular rate and rhythm, temporary venous pacemaker VVI set to 80 beats per minute. Radial and femoral pulses intact |
| **Abdomen** | Abdomen distended and diffusely tender with severe guarding. Hypoactive bowel sounds. PEG tube in place to dependent drainage. |
| **Neurological** | Sedated, Richmond Agitation-Sedation Scale (RASS) -1. Decreased strength in right upper and lower extremities. |
| **Skin** |  |
| **GU** |  |
| **Psychiatric** |  |
