# Supplementary material for: Anesthesiology Handoff Simulation Case: A Handoff From Intensive Care Unit to Operating Room for Anesthesiology Learners
Source: MedEdPORTAL. 2020 Mar 13;16:10887. doi: 10.15766/mep_2374-8265.10887 (PMC7083603; doi:10.15766/mep_2374-8265.10887)
Supplement: Supplementary file 1 — A. Simulation Case.docx B. Learner Case.docx C. Scoring Key.docx D. Teaching Points.docx E. Learner Evaluation.docx [file mep-16-10887-s001.zip › C. Scoring Key.docx]

Appendix C

Quantitative Scoring Key

|  | Points available |
| --- | --- |
| **Surgical procedure to be done** | /1 |
| **Past medical history** | /1 |
| **Neurologic deficits** | /1 |
| **Ventilator settings** | /1 |
| **Current airway status** |  |
| Trach/ETT/cuffed/uncuffed | /1 |
| Concern over airway edema/fluid overload | /1 |
| **NPO status** | /1 |
| **Recent PCI/stenting** |  |
| BMS/DES | /1 |
| Anticoagulation | /1 |
| Last dose | /1 |
| INR | /1 |
| **ICD/pacemaker settings** | /1 |
| **Current rhythm** | /1 |
| **Anti-hypertensive medications** |  |
| Beta blocker/ACEi/diuretic | /1 |
| **Echocardiogram** |  |
| LVEF mention | /1 |
| Valves, RV dilatation, potential dysfunction | /1 |
| **Transfusions** |  |
| Blood products given/hold for OR | /1 |
| Specific mention of pulmonary congestion post fluid/transfusion therapy early in admission | /1 |
| **Discussion of laboratory values (recent electrolytes, ABG, etc.)** | /1 |
| **History of diabetes mellitus** | /1 |
| Current blood glucose | /1 |
| Treatment | /1 |
| **Last dialysis received/fluid removed** | /1 |
| **Antibiotics** |  |
| Scheduled dosing | /1 |
| Days given | /1 |
| **Vascular access** |  |
| Peripheral IV’s: size and site | /1 |
| Central line: Type, size and side | /1 |
| **Sign out completed within time limit** | /1 |
| TOTAL | /28 |

Qualitative Scoring Key

| **Core**  **Competency** | **Requires constant guidance**  **(1)** | **Requires frequent guidance**  **(2)** | **Requires occasional guidance**  **(3)** | **Ready to transition into independent practice (4)** |
| --- | --- | --- | --- | --- |
| **Patient care** | Identifies few medical issues and has minimal understanding of anesthetic implications | Identifies some relevant medical issues, and understands some anesthetic implications | Identifies almost all relevant medical issues, and understands most anesthetic implications | Identifies all relevant medical issues, and understands all anesthetic implications |
|  |  |  |  |  |
| **Practice-based learning and improvement** | Is unable to explain basic anesthesia concepts to other health professionals | Effectively explains basic anesthesia concepts to other health professionals | Effectively explains anesthesia concepts to other health professionals | Serves as an expert on anesthesia concepts |
|  |  |  |  |  |
| **Professionalism** | Does not display compassion, respect, and empathy for patients | Displays compassion, respect, and empathy for the patients for some of the handoff with major lapses | Displays compassion, respect, and empathy for patients for the majority of the handoff with minor lapses | Displays compassion, respect, and empathy for patients, acts as a reliable team member |
|  |  |  |  |  |
| **Interpersonal and communication skills** | Never communicates challenging information in a concise and easy to understand manner | Occasionally communicates challenging information in a concise and easy to understand manner | Mostly communicates challenging information in a mostly concise and easy to understand manner | Communicates challenging information in a concise and easy to understand manner |
|  |  |  |  |  |
