# Supplementary material for: Anesthesiology Handoff Simulation Case: A Handoff From Intensive Care Unit to Operating Room for Anesthesiology Learners
Source: MedEdPORTAL. 2020 Mar 13;16:10887. doi: 10.15766/mep_2374-8265.10887 (PMC7083603; doi:10.15766/mep_2374-8265.10887)
Supplement: Supplementary file 1 — A. Simulation Case.docx B. Learner Case.docx C. Scoring Key.docx D. Teaching Points.docx E. Learner Evaluation.docx [file mep-16-10887-s001.zip › D. Teaching Points.docx]

Appendix D

Teaching Points

The purpose of this document is to provide a summary and analysis of the key medical issues and anesthetic implications that are essential for a complete handoff in this simulation. This can be used by the facilitator as a refresher prior to the simulation, it can be used to provide information to the resident about points that they may have missed, and it can even be given to the learner to peruse following the simulation. These subjects were chosen to be included in our simulation because they are commonly occurring medical issues in critically ill patients presenting to the operating room and are critical components to report during an anesthesia handoff.

**Surgical procedure**

Basic knowledge of the patient and the procedure is essential for the anesthetic plan. For example, this patient is undergoing urgent exploratory laparotomy, and, as such, learners should be thinking about peripheral IV access, the need for potential transfusion, and even possible arterial line placement for hemodynamic monitoring due to patient's coronary artery disease/congestive heart failure and risk for blood loss.

**Complete past medical history**

A thorough history allows for the planning of safe and proper anesthesia care. Again, basic knowledge of the patient and her comorbidities can potentially alter anesthetic management significantly. Even something as simple as a pre-operative blood sugar in a diabetic patient is important to know. Knowledge of this can allow the anesthesia team to plan for intraoperative and post-operative glucose management. Research has shown a clear association with hyperglycemia and adverse clinical outcomes [1,2,3].

**Neurologic deficits**

Neurologic deficits in a subdural hematoma can be variable and often depend on the quantity of bleeding and ensuing increases in intracranial pressure (ICP) and midline shift. Acutely increasing size of bleeding following traumatic head injury can lead to mass effect in the brain and may require emergent craniotomy for decompression [4,5,6]. Establishing baseline deficits also becomes especially important in patients going to the OR after subarachnoid hemorrhages, as their risk for cerebral arterial vasospasm in addition to increased ICP issues is significant, and specific management options need to be considered [7].

**Current ventilator settings**

Handing off current ventilator settings are important to explain the respiratory status of a patient undergoing anesthesia. Our patient’s ventilator settings are given in the clinical vignette along with the most recent arterial blood gas. In our patient’s situation, it may be necessary for the operative team to make changes in her settings to improve both her acid-base status and her oxygenation. Employment of lung protective ventilation strategies such as low tidal volume ventilation through the perioperative period is indicated in ICU patients to decrease their risk of barotrauma or volutrauma and lung injury [8,9].

**Current airway status**

Understanding the airway status of a patient is a critical part of an anesthesia handoff. Information about a patient’s airway can include Mallampati Score, atlanto-occipital joint extension, thyromental distance, sternomental distance, mandibulo-hyoid distance, and inter-incisor distance [10]. Reporting the type of endotracheal tube (size) or tracheostomy (Fenestrated/Unfenestrated, cuffed/uncuffed) will affect inhalational anesthetic delivery and ventilator management and thus is essential to handoff to the anesthesia provider [11].

**NPO status**

The risk of aspiration during anesthetic induction is increased in patients with fatty food ingestion <8 hours, diabetic gastroparesis, bowel obstruction, significant gastroesophageal reflux disease, pregnant women in the second and third trimesters, upper GI bleeding, among many others [12]. Aspiration of solid matter can lead to hypoxia due to airway obstruction, and aspiration of acidic content can cause a pneumonitis leading to acute lung injury, hypoxia, and hemodynamic instability [12,13]. The risk of mortality and serious morbidity increases with bronchial exposure to greater volumes and lower acidity of aspirated material. As little as 50ml of acidic gastric content can be considered to be severe aspiration and can lead to significant morbidity for the patient [13]. Current ASA guidelines indicate that patients ingesting clear liquids should have a fasting time of 2 hours. A fast of 6 hours is recommended for a light meal and, if fatty foods are ingested, a fast of 8 hours is recommended [14].

**Recent percutaneous coronary intervention (PCI)/stenting**

Recent stenting can be a contraindication for elective surgery within specific time frames based on the type of stent placed (drug-eluting or bare metal) and their respective anticoagulation regimens. Patients with drug-eluting stents should have elective surgery postponed for at least 6 months. If a bare metal stent has been placed, 30 days must pass prior to any elective procedure. If urgent surgery is required, antiplatelet consideration must be discussed [15]. Our patient is going for *urgent surgery* and not elective surgery, but her recent drug-eluting stent and anticoagulation status are still essential to discuss with the operative team during the handoff.

**ICD/pacemaker settings**

Patients with cardiovascular electronic implantable devices (CEIDs) often have underlying cardiac disease that includes structural heart disease, chronic heart failure, or inherited arrhythmia syndromes [16]. Preoperative evaluation of these patients should encompass an awareness of the device and the type of cardiac device being employed [16,17]. The Heart Rhythm Society along with the American Society of Anesthesiologists have issued an expert consensus statement that acknowledges that because of the complexity and variety of indications, a single recommendation for all patients is not appropriate and should be individualized [16]. In our patient, the use of VVI pacing has been employed for 3^rd^ degree AV block, and should be continued throughout the procedure. Bipolar electrocautery should be employed if available [17].

**Current rhythm**

Preoperative resting 12-lead EKG is reasonable for patients with known coronary artery disease, significant arrhythmia, peripheral artery disease, cerebrovascular disease, or other significant structural heart disease (Class IIa) [16]. If a recent EKG is available, its findings should be conveyed to the anesthesia provider during the handoff. Not only does the EKG contain information relating to short and long-term morbidity and mortality it may provide a useful baseline standard to measure changes in the postoperative period [16].

**BP medications/beta blockers**

Hypertension is one of the leading causes of morbidity worldwide [18]. Hypertensive urgency is defined as a systolic BP >180 and diastolic >110 [18]. In one study, no significant difference in outcomes were seen in patients that underwent treatment and proceeded to the OR versus rescheduling surgery [19]. However, recommendations for postponing elective surgery in the setting of hypertensive urgency remain, especially in the setting of other cardiovascular comorbidities [18]. No antihypertensive medications are discontinued in the preoperative time frame, with the exception of angiotensin converting enzyme inhibitors (ACEis) and angiotensin II receptor blockers (ARBs). Current findings indicate withholding ACEis and ARBs for 24 hours prior to undergoing major non-cardiac surgery is associated with a lower risk of death and postoperative vascular events [20]. Larger randomized controlled trials are still needed, however, to confirm these findings.

**Echocardiography**

Routine use of LV function testing for patients undergoing elective non-cardiac surgery is currently not recommended [16]. Current recommendations for preoperative echocardiography are strongest for those with new-onset dyspnea of unknown origin and those with an existing history of heart failure with worsening dyspnea or other clinical symptoms [16]. In the case of our patient, an echocardiogram already exists with documented reduced left ventricular ejection fraction (LVEF) in the setting of coronary artery disease. Studies have demonstrated an association between reduced LVEF and perioperative complications [16]. There is an association between the degree of systolic dysfunction and risk of complications, with the greatest risk seen in patients with an LVEF <35% at rest [16].

**Transfusions**

Transfusion-associated lung injury and acute hemolytic transfusion reactions are just two of many complications associated with the transfusion of blood products leading to significant morbidity and mortality. Studies have shown no improvement in outcomes with liberal transfusion strategies [21]. Thus, it becomes prudent to use more objective measures in the intraoperative setting to assess for the need for transfusion of blood products, such as hemoglobin/hematocrit values [21].

**Electrolytes**

Severe electrolyte abnormalities should be corrected prior to undergoing surgery. As patients age their predisposition for developing hypo- and hyperkalemia increases, which can be complicated by their use of diuretics [22]. Hyperkalemia is defined as serum concentration greater than 5.5 mEq and has typically been considered as a relative contraindication to the use of succinylcholine as a paralytic [23]. In critically-ill patients with prolonged immobilization, significant muscular atrophy can lead to a severe hyperkalemic crisis with the use of succinylcholine and should generally be avoided [24].

**History of diabetes mellitus**

Surgery and general anesthesia cause a neuroendocrine stress response with release of counterregulatory hormones such as epinephrine, glucagon, cortisol, and growth hormone [25]. These changes can result in metabolic abnormalities, insulin resistance, decreased peripheral glucose utilization, and impaired insulin secretion. This can lead to hyperglycemia and ketosis [25]. Perioperative glycemic control includes avoidance of hypoglycemia, prevention of ketoacidosis or hyperosmolar states, maintenance of fluid and electrolyte balance, and avoidance of marked hyperglycemia [25,26]. It is recommended that oral hypoglycemics and non-insulin injectable drugs be held; sulfonylureas increase the risk of hypoglycemia, metformin increases the risk of renal hypoperfusion and lactate accumulation [25,26]. Insulin should be monitored every 2 hours via fingerstick and treated with insulin [25]. The American Diabetes Association endorses a target glucose range for the perioperative period of 80 – 180 mg/dL [25].

**Last dialysis**

There is a higher perioperative mortality rate in the ESRD population than the non-ESRD population [27]. If the surgery is non-elective, the benefit of emergent dialysis will need to be considered against the patient’s clinical condition and the ability to tolerate ultrafiltration. No guidelines exist at present which define a safe limit to the potassium level prior to anesthesia induction [28]. If dialysis is held prior to surgery, the risk of uremia associated bleeding and hyperkalemia needs to be considered [28].

**Antibiotics**

The use of preoperative antibiotics to decrease the risk of post-operative infection has been well documented in the literature [29]. The world health organization has released extensive guidelines on multiple prevention modalities, including antibiotic selection and appropriate timing for effective prophylaxis [30].

**Vascular access**

Venous access is required for intravenous drug administration and should be present in all patients proceeding to the operating room for major, urgent surgery. Certain considerations must be present in the location and quality of vascular access, such as end stage renal disease patients [27]. Critically ill patients often require invasive hemodynamic monitoring and larger vascular access to provide aggressive fluid and vasopressor support [31]. Identifying available hemodynamic monitors and intravenous access available to the anesthesia provider will help improve patient care in the operating room.

**References:**

1. Umpierrez G, Isaacs S, Bazargan N, et al. Hyperglycemia: An independent marker of in-hospital mortality in patients with undiagnosed diabetes. *J Clin Endocrinol Metab*. 2002;87:978-82.
2. Frisch A, Chandra P, Smiley D, Peng, L, et al. Prevalence and clinical outcome of hyperglycemia in the perioperative period in noncardiac surgery. *Diabetes Care*. 2010;33:1783-8.
3. Kotagal M, Symons R, Hirsch I, et al. Perioperative hyperglycemia and risk of adverse events among patients with and without diabetes. *Ann Surg*. 2015;261:97-103.
4. Mulligan P, Raore B, Liu S, et al. Neurological and functional outcomes of subdural hematoma evacuation in patients over 70 years of age. *Journal of Neurosciences in Rural Practice*. 2013;4(3):250-256.
5. Karibe H, Hayashi T, Hirano T, et al. Surgical management of traumatic acute subdural hematoma in adults: a review. *Neurologia Medico-Chirurgica*. 2014;54(11):887=894.
6. Bullock M, Chesnut R, Ghajar J, et al. Surgical management of acute subdural hematomas. Neurosurgery. 2006;58(3)16-24.
7. Sriganesh K, Venkataramaiah S. Concerns and challenges during anesthetic management of aneurysmal subarachnoid hemorrhage. *Saudi Journal of Anaesthesia*. 2015;9(3):306-313.
8. Ochiai R. Mechanical ventilation of acute respiratory distress syndrome. *Journal of Intensive Care.* 2015;3(1)25.
9. Salman D, Finney S, Griffiths M. Strategies to reduce ventilator-associated lung injury (VALI). *Burns.* 2013;39(2):200-211.
10. Crawley S, Dalton A. Predicting the difficult airway. Continuing Education in Anaesthesia Critical Care & Pain. 2015;15(1):253-257.
11. Regan K, Hunt K. Tracheostomy management. Continuing Education in Anaesthesia Critical Care & Pain. 2008;8(1):31-35.
12. Robinson M, Davidson A. Aspiration under anaesthesia: risk assessment and decision-making. Continuing Education in Anaesthesia Critical Care & Pain. 2014;14(4):171-175.
13. Nason K. Acute Intraoperative Pulmonary Aspiration. *Thoracic Surgery Clinics*. 2015;25(3):301-307.
14. Practice Guidelines for Preoperative Fasting and the Use of Pharmacologic Agents to Reduce the Risk of Pulmonary Aspiration: Application to Healthy Patients Undergoing Elective Procedures: An Updated Report by the American Society of Anesthesiologists Task Force on Preoperative Fasting and the Use of Pharmacologic Agents to Reduce the Risk of Pulmonary Aspiration. *Anesthesiology*. 2017;126:376-393.
15. Duration of Dual Antiplatelet Therapy: A Systematic Review for the 2016 ACC/AHA Guideline Focused Update on Duration of Dual Antiplatelet Therapy in Patients With Coronary Artery Disease: A Report of the American College of Cardiology/American Heart Association Task Force on Clinical Practice Guidelines. *Circulation.* 2016;134(10):1116-1139.
16. Fleisher L, Fleischmann K. 2014 ACC/AHA Guideline on Perioperative Cardiovascular Evaluation and Management of Patients Undergoing Noncardiac Surgery. *Journal of the American College of Cardiology*. 2014;64(22):77-137.
17. Crossley G, Poole J, Rozner M, et al. The Heart Rhythm Society (HRS)/American Society of Anesthesiologists (ASA) Expert Consensus Statement on the Perioperative Management of Patients with Implantable Defibrillators, Pacemakers and Arrhythmia Monitors: Facilities and Patient Management. *Heart Rhythm.* 2011;8(7):1114-1154.
18. Varon J, Marik, P. Perioperative hypertension management. *Vascular Health and Risk Management*. 2008;4(3):615-627.
19. Weksler N, Klein M, Szendro G, et al. The dilemma of immediate preoperative hypertension: to treat and operate, or to postpone surgery? J Clin Anesth. 2003;15:179-183.
20. Roshanov P, Rochwerg B, Patel A, et al. Withholding versus Continuing Angiotensin-converting Enzyme Inhibitors or Angiotensin II Receptor Blockers before Noncardiac Surgery: An Analysis of the Vascular events In Noncardiac Surgery Patients Cohort Evaluation Prospective Cohort. Anesthesiology. 2017;126(1):16-27.
21. Practice Guidelines for Perioperative Blood Management: An Updated Report by the American Society of Anesthesiologists Task Force on Perioperative Blood Management. Anesthesiology. 2015;122(2):241-275.
22. Butterworth J, Mackey D, Wasnick J. “Geriatric Anesthesia.” In: *Morgan and Mikhail’s Clinical Anesthesiology.* 5th ed. McGraw-Hill; 2013:907-917.
23. Schow M, Lubarsky A, Olson D, et al. Can succinylcholine be used safely in hyperkalemic patients? *Anesthesia and Analgesia*. 2002;95(1):119-122.
24. Martyn J, Richtsfeld M. Succinylcholine-induced Hyperkalemia in Acquired Pathologic States: Etiologic Factors and Molecular Mechanisms. Anesthesiology. 2006;104(1):158-169.
25. Duggan E, Carlson K, Umpierrez G. Perioperative Hyperglycemia Management: An Update. Anesthesiology. 2017;126(3):547-560.
26. Joshi G, Chung F, Vann M, et al. Society for Ambulatory Anesthesia consensus statement on perioperative blood glucose management in diabetic patients undergoing ambulatory surgery. *Anesth Analg.* 2010;111:1378.
27. Nasr R, Chilimuri S. Preoperative Evaluation in Patients With End-Stage Renal Disease and Chronic Kidney Disease. *Health Services Insights.* 2017;10.
28. Sanghani N, Soundararajan R, Weavind L, et al. Medical Management of the dialysis patient undergoing surgery. *UpToDate*.
29. Leaper D, Burman-Roy S, Palanca A, et al. Prevention and treatment of surgical site infection: summary of NICE guidance. *BMJ.* 2008;337:1924.
30. *Global Guidelines for the Prevention of Surgical Site Infection*. (2017). World Health Organization.
31. Huygh J, Peeters Y, Bernards J, et al. Hemodynamic monitoring in the critically ill: an overview of current cardiac output monitoring methods. *F1000Research.* 2016;5:F1000.
